# Advances in the role of the GADD45 family in neurodevelopmental, neurodegenerative, and neuropsychiatric disorders

**DOI:** 10.3389/fnins.2024.1349409

**Published:** 2024-01-25

**Authors:** Mengbing Huang, Ji Wang, Wei Liu, Hongyan Zhou

**Affiliations:** Hubei Key Laboratory of Cognitive and Affective Disorders, Institute of Biomedical Sciences, School of Medicine, Jianghan University, Wuhan, China

**Keywords:** GADD45 family, DNA demethylation, neurodevelopmental disorders, neurodegenerative diseases, neuropsychiatric disorders

## Abstract

The growth arrest and DNA damage inducible protein 45 (GADD45) family comprises stress-induced nuclear proteins that interact with DNA demethylases to facilitate DNA demethylation, thereby regulating diverse cellular processes including oxidative stress, DNA damage repair, apoptosis, proliferation, differentiation, inflammation, and neuroplasticity by modulating the expression patterns of specific genes. Widely expressed in the central nervous system, the GADD45 family plays a pivotal role in various neurological disorders, rendering it a potential therapeutic target for central nervous system diseases. This review presented a comprehensive overview of the expression patterns and potential mechanisms of action associated with each member of GADD45 family (GADD45α, GADD45β, and GADD45γ) in neurodevelopmental, neurodegenerative, and neuropsychiatric disorders, while also explored strategies to harness these mechanisms for intervention and treatment. Future research should prioritize the development of effective modulators targeting the GADD45 family for clinical trials aimed at treating central nervous system diseases.

## 1 Introduction

### 1.1 GADD45 family

The GADD45 family comprises a group of stress-responsive nuclear proteins, consisting of three members: GADD45α, GADD45β, and GADD45γ. The structure of GADD45 family genes in humans and mice are similar. The *Gadd45α* gene is located on human chromosome 1p31.3 (mice chromosome 6 C1; 6 30.72 cM), encoding 165 amino acids, with a protein size of 18kD and localized in the cell nucleus. The *Gadd45β* gene is situated on human chromosome 19p13.3 (mice chromosome 10 C1; 10 39.72 cM), encoding 160 amino acids, with a protein size of 18kD, located in both the cell nucleus and extracellular matrix. The *Gadd45γ* gene is found on human chromosome 9p22.2 (mice chromosome 13 A5; 13 26.36 cM), encoding 159 amino acids, with a protein size of 17kD and in both the cell nucleus and extracellular matrix.^1^

Serving as prompt sensors for cellular and environmental damage, GADD45 family proteins actively participate in the regulation of cell cycle progression, DNA damage repair mechanisms, programmed cell death pathways, and control of gene expression levels. Consequently, they contribute to diverse cellular processes (Schmitz, 2022). Research findings suggest that p53 has the ability to induce the expression of GADD45α and GADD45β, thereby leading to cell cycle arrest or apoptosis (Mo et al., 2021). Moreover, a positive regulatory loop is formed between GADD45α and GADD45β, which positively regulates p53. The interaction of the GADD45 family with MTK1/MEKK4, an upstream kinase of MAPK, activates the JNK and p38 signaling pathways, consequently influencing crucial cellular processes such as proliferation, differentiation, migration, and apoptosis (Liao et al., 2020). Additionally, it has been observed that GADD45β can bind to the NF-κB subunit p65 and facilitate its nuclear localization as well as transcriptional activity. Furthermore, through its interaction with IKKβ protein, GADD45γ exhibits inhibitory effects on NF-κB activation (Wang et al., 2005).

### 1.2 GADD45 family and DNA methylation

**Figure 1:**
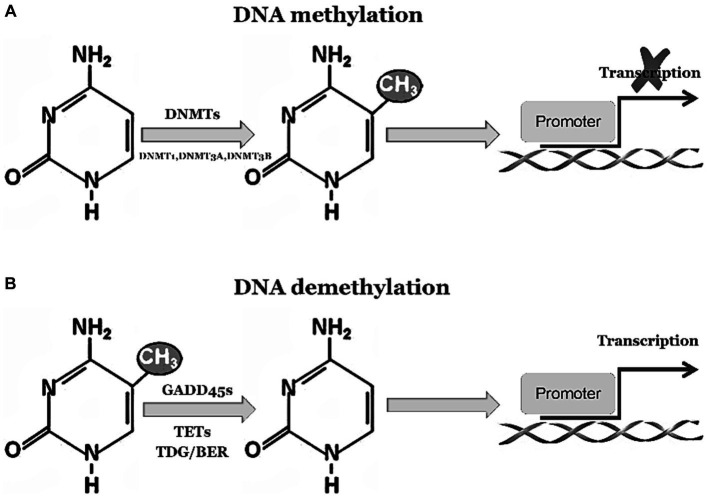
GADD45 family participates in DNA demethylation process. **(A)** 5-Methylcytosine (5mC) modification is a stable suppressive regulatory factor in gene expression, predominantly found within CpG islands in gene promoter regions, representing the most abundant and widespread DNA modification in the human genome. 5mC is integrated into DNA by the methyltransferases DNMT3a and DNMT3b. Subsequently, during DNA replication, the methyltransferase DNMT1 replicates these methylated marks onto the daughter strands, ensuring the maintenance of methylation marks throughout the entire genome. **(B)** 5mC undergoes oxidation by the Tet family proteins to generate 5-hydroxymethylcytosine (5hmC) and 5-carboxylcytosine (5caC). The DNA glycosylase TDG/BER removes 5caC, initiating base excision repair pathways to accomplish DNA demethylation. The activation or inhibition of demethylation relies on the interaction between Tet-TDG and the GADD45 family.

The GADD45 family is closely associated with DNA demethylation, playing a crucial role in facilitating this process (You et al., 2019). DNA demethylation is an epigenetic regulatory mechanism that involves the removal of methyl groups from DNA molecules, thereby influencing gene expression. Under normal circumstances, activation of GADD45 family proteins occurs in response to environmental stressors or challenging conditions. These stressors can include factors such as DNA damage, inflammation, and exposure to chemical substances (Humayun and Fornace, 2022). Activated GADD45 family proteins interact reciprocally with DNA demethylases to promote the process of DNA demethylation. Consequently, specific genes undergo altered expression patterns through activation or repression of particular genetic elements. These events have profound effects on cellular functionality and epigenetic regulation, as depicted in Figure 1. In summary, the involvement of GADD45 family proteins is essential for efficient DNA demethylation processes and significantly contributes to modulating gene expression patterns while maintaining cellular stability under various environmental stressors and challenging conditions. Their engagement plays a paramount role in ensuring normal physiological cell functions as well as disease prevention and regulation.

### 1.3 Effects of GADD45 family on central nervous system diseases

Neurological disorders encompass a range of neurological abnormalities that can be broadly categorized into three groups: (1) neurodevelopmental disorders, such as autism spectrum disorders (ASD), schizophrenia (SCZ), and attention deficit hyperactivity disorder (ADHD); (2) neurodegenerative disorders, including Alzheimer’s disease (AD), Parkinson’s disease (PD), and amyotrophic lateral sclerosis (ALS); (3) neuropsychiatric disorders, such as major depression disorder (MDD), post-traumatic stress disorder (PTSD), bipolar disorder (BD), and addiction disorder (ADD). Their etiology and pathophysiology often overlap GADD45-dependent molecular signaling pathways. For example, GADD45α exhibits elevated expression in the 1-methyl-4-phenylpyridine (MPP^+^)-induced *in vitro* model of PD, with a notable exacerbation of cell damage upon GADD45α downregulation (Wang et al., 2014). In the temporal gyrus of individuals with ASD, the expression level of GADD45β is significantly elevated (Garbett et al., 2008). Conversely, in the prefrontal cortex of individuals with SCZ, the expression level of GADD45β is aberrantly diminished (Gavin et al., 2012). Notably, in the substantia nigra of individuals with PD, GADD45β manifests an abnormally heightened expression pattern (Yang et al., 2016). Moreover, mice with GADD45γ gene defects exhibit pronounced cognitive dysfunction and depressive-like behavior (Zhang et al., 2022). The investigation of the GADD45 family in the aforementioned neurological disorders has been growing, and there exists substantial heterogeneity in the expression and functionality of each family member across different diseases. Despite reports on the expression patterns and mechanisms of GADD45β in neurological disorders, a comprehensive synthesis and analysis of the entire GADD45 family, particularly GADD45α and GADD45γ, across diverse diseases is still lacking. Here, we will explore the expression patterns, diverse functions and molecular pathways regulated by GADD45 family members in neurodevelopmental, neurodegenerative, and neuropsychiatric disorders. We will discuss (1) how modulation of GADD45 expression in nerve and glial damage, oxidative stress, DNA damage repair, apoptosis, proliferation, differentiation, inflammation, and neuroplasticity; (2) how studies in human patient tissues, as well as *in vitro* and *in vivo* model systems, have uncovered the roles of GADD45 in central nervous system diseases.

## 2 GADD45 family in neurodevelopmental disorders

Neurodevelopmental disorders refer to behavioral and cognitive impairments that manifest during the developmental process, leading to noticeable difficulties in learning and mastering certain intellectual, motor, or social skills. Approximately 15–20% of children under 18 years of age are diagnosed with a developmental disability in the United States (Francés et al., 2022). The therapeutic landscape for neurodevelopmental disorders, including ASD, SCZ, and ADHD, remains constrained (Baj et al., 2021).

Research indicates that GADD45β exhibits relatively weak expression in mouse neural tissues, while GADD45α demonstrates low expression in the embryonic or early postnatal CD1 mouse brain and in the fetal forebrain during pregnancy (Kaufmann and Niehrs, 2011; Xiao et al., 2021). Both *in vitro* and *in vivo* studies reveal that both knockdown and overexpression of GADD45α significantly impair neuronal morphology, reduce the complexity of neuronal synapses, and disrupt neuronal differentiation, suggesting a crucial role for GADD45α expression in the development of mouse neural circuits (Sarkisian and Siebzehnrubl, 2012). Conversely, GADD45γ attains its highest expression in neural progenitor cells in mice and frogs, implying its potential regulatory role in the growth and development of both central and peripheral nervous systems (Kaufmann et al., 2011). These findings suggest a potential role for GADD45 family genes in embryonic and neural development. Further investigation, through siRNA-mediated knockdown of GADD45β expression in the amygdala of neonatal rats, revealed that GADD45β knockdown altered the social behavior of neonatal rats while concurrently reducing the expression of several genes associated with psychiatric disorders. These genes include Methyl-CpG-binding protein 2 (MeCP2), extracellular glycoprotein Reelin, and brain-derived neurotrophic factor (BDNF) (Kigar et al., 2015). This implies that GADD45β plays a crucial role in the epigenetic processes of complex adolescent social interactions and may offer insights into the etiology of adolescent behavioral disorders. Subsequently, we will focus on elucidating the impact of the GADD45 family on neurodevelopmental disorders such as ASD, SCZ, and ADHD, detailing potential signaling pathways and summarizing the expression patterns of GADD45 family proteins.

### 2.1 GADD45 family and ASD

ASD is a pervasive developmental disorder characterized by communication difficulties, language impairments, and atypical behaviors. Its core symptoms include social impairments, language difficulties, and repetitive stereotyped behaviors, commonly referred to as the “triad of impairments.” From a genetic perspective, hundreds of genes associated with ASD development have been identified (Satterstrom et al., 2020). This indicates that ASD is not a simple monogenic disorder but rather a complex polygenic condition. The World Health Organization estimates the worldwide prevalence of ASD at 0.76%. Therefore, ASD affects approximately 16% of the global child population (Baxter et al., 2015). Zhou et al. (2017) conducted a 4-year epidemiological survey of ASD in Chinese school-age children, revealing, for the first time, the epidemiological characteristics of ASD in this population. The study found a prevalence rate of 0.7% for ASD among Chinese school-age children, with a male-to-female ratio of 4.1:1, highlighting the challenging landscape for the prevention and management of ASD in China. According to the 2023 Centres for Disease Control and Prevention (CDC) report, the United States prevalence of ASD is 1 in 36 children, it is higher in boys than in girls and is continuously increasing (Christensen et al., 2016; Shaw et al., 2023). The cause of ASD is unknown, and its symptoms can persist into adulthood, accompanied by physical impairment and disability, making its diagnosis and treatment complex. A foreign study analyzing blood samples from 1,263 newborns found that the risk of children developing ASD might be linked to the methylation levels of certain DNA in the genome (Hannon et al., 2018). This implies that epigenetics, especially DNA methylation, plays a crucial role in the development of ASD. Therefore, it is reasonable to suspect that the GADD45 family, closely related to DNA demethylation, may play a pivotal role in the developmental processes of ASD.

Clinical studies have revealed the presence of GADD45α gene deletion in peripheral blood T cells of 6-year-old children with severe language impairments (Tassano et al., 2015). Conversely, ASD patients exhibit a significant increase in the expression levels of GADD45β in the temporal gyrus of the brain (Garbett et al., 2008). The differential expression of GADD45 family members in various locations indicates functional heterogeneity within the GADD45 family. Research also highlights disruptions in lymphocyte DNA methylation levels and frontal cortex neuron histone methylation levels in ASD patients (Nguyen et al., 2010). Furthermore, there is a notable enrichment of the ASD-associated epigenetic signal H3K4me3 histone modification in the regions surrounding gene transcription start sites related to neurodevelopmental disorders (Shulha et al., 2012). In the brain tissues of ASD subjects, DNA methylation has been identified in the upstream promoter regions of retinoic acid-related orphan receptor alpha (RORA), a nuclear receptor associated with multiple gene networks and neurotransmitter pathways. This methylation leads to a decrease in RORA protein expression levels (Nguyen et al., 2010), potentially associated with insufficient levels of GADD45β in brain tissues. In animal experiments, the BTBRT+Itpr3tfJ (BTBR) inbred mouse strain is recognized as one of the most stable animal models reflecting the core clinical features of ASD. Researchers assessed genetic and epigenetic changes in BTBR mouse brain tissues using an improved comet assay and found increased oxidative DNA strand breaks and overall DNA methylation levels (5-mC%) in bone marrow cells of BTBR mice. Additionally, the mRNA expression levels of GADD45α and GADD45γ were elevated in the hippocampal tissues of BTBR mice (Attia et al., 2020). After contextual fear conditioning and exposure, the expression levels of GADD45β mRNA in the hippocampal tissues of mice were also higher than those in the control group, suggesting that GADD45β plays a crucial role in hippocampus-dependent long-term memory formation, possibly through the promotion of DNA demethylation (Leach et al., 2012). Furthermore, in the brain C1 region tissues of C57BL/6 mice undergoing contextual fear conditioning, there was a significant increase in the expression levels of GADD45β and GADD45γ mRNA (Sultan et al., 2012). Although there is functional heterogeneity among GADD45 family members, considering the fundamental structural functions of the GADD45 family and the physiological functions of downstream target genes, we hypothesize that the GADD45 family is underexpressed in ASD, playing a role in the regulation of DNA methylation levels, thereby influencing central nervous system function in the brain and participating in the development of ASD.

### 2.2 GADD45 family and SCZ

Clinical features of SCZ include patients experiencing hallucinations, delusions, disorganized thinking, and emotional disturbances, with significant disruptions in social and cognitive functions and severe impairments in reality perception. This disorder primarily emerges in late adolescence or early adulthood, profoundly affecting the thoughts, emotions, and behaviors of individuals. In terms of pathological characteristics, SCZ patients exhibit abnormal neurotransmitter function, particularly in the dopamine (DA) system. *In vivo* imaging studies confirm a significant increase in striatal DA synthesis and release capacity in patients (Bellon et al., 2022). Brain imaging studies also indicate a reduction in intracranial volume in SCZ patients (Barth et al., 2021). Additionally, genetic factors play a role in disease risk, with individuals having a family history of SCZ being more susceptible to its impact (Zhang et al., 2023).

Scientists studying the biological mechanisms of SCZ have discovered significant differences in DNA methylation levels of the BDNF gene in frontal cortex biopsy tissues of SCZ patients compared to the control group (Mill et al., 2008). In the frontal cortex of healthy individuals, both excitatory and inhibitory cortical neurons express GADD45β, while glial cells do not express GADD45β (Gavin et al., 2012). However, in the frontal cortex layers II, III, and V of individuals with psychiatric disorders (including 15 cases of SCZ and 11 cases of BD), there is an increase in GADD45β-positive cells. This is associated with reduced binding to the BDNF promoter, increased expression of 5-methylcytosine and 5-hydroxymethylcytosine at the promoter, leading to decreased BDNF mRNA expression (Gavin et al., 2012). This suggests that the increased presence of GADD45β in psychiatric patients may be a compensatory response to a highly methylated cellular environment. Additionally, GADD45 proteins are implicated in the pathological processes and histone modifications during the treatment SCZ (Guidotti et al., 2009; Labrie et al., 2012). In animal investigations, intraperitoneal administration of 0.5 mg/kg LY379268 (mGlu2/3 agonist) to Swiss mice resulted in enhancement expression of GADD45β and GADD45γ mRNA, as well as heightened protein expression of GADD45β within the frontal cortex. Additionally, LY379268 elicited an elevation in the expression levels of BDNF and glutamic acid decarboxylase-67 (GAD67), both binding to specific promoter regions (Matrisciano et al., 2011). The findings of these studies suggest that GADD45β and GADD45γ exert an influence on neurosignal alterations within the brains of SCZ individuals and mediate the effects of certain therapeutic modalities for SCZ. It is noteworthy that GADD45β in SCZ may selectively propel an upregulation in the expression levels of genes associated with BDNF, indicating a potential regulatory role of GADD45β in the demethylation process of critical genes implicated in central nervous system disorders. This observation suggests that members of the GADD45 gene family might emerge as prospective targets for the treatment of SCZ.

### 2.3 GADD45 family and ADHD

ADHD is a childhood neurodevelopmental disorder characterized by persistent deficits in attention, hyperactivity, and impulsive behavior. The prevalence of ADHD in the pediatric population ranges from 5 to 10% (Castellano-García and Benito, 2022). Primarily instigated by genetic factors, the heritability of this condition reaches an estimable 74% (Faraone and Larsson, 2019). Furthermore, various environmental factors, such as early nutritional deficiencies, birth complications, infections, exposure to toxins, and stress, may exert influence on the onset and progression of ADHD (Doi et al., 2022). In the realm of genetics, DNA methylation, functioning as a genomic epigenetic regulatory mechanism, has been discerned as a participant in certain pathogenic mechanisms underlying ADHD.

The investigation elucidates a correlation between the methylation levels of the cg25520701 locus within the cyclic-AMP response binding protein 5 (CREB5) gene in umbilical cord blood during pregnancy and the diagnosis of ADHD (Neumann et al., 2020). Sequencing results targeting specific genes (such as DRD4, KLDR1, and TARBP1) for DNA methylation sites in whole blood DNA of children or adults with ADHD, indicate that certain sites are associated with the adult ADHD state. Moreover, multiple DNA methylation sites within TAR RNA binding protein 1 (TARBP1) exhibit correlation with symptoms of both adult and pediatric ADHD (Weiß et al., 2021). This suggests that DNA methylation, as a crucial mechanism of epigenetics, plays a significant role in the development and persistence of ADHD. The GADD45 family, as specific gene promoter DNA demethylating agents, plays a crucial role in neurodevelopment. For instance, knockout of GADD45β alters the social behavior of neonatal rats, concomitantly reducing the expression levels of genes associated with psychiatric disorders, including MeCP2, Reelin, and BDNF (Kigar et al., 2015). Consequently, GADD45β assumes a significant role in the epigenetic programs of complex adolescent social interactions, concurrently offering valuable insights into the etiology of adolescent behavioral disorders such as ADHD, ASD, and/or SCZ.

**Figure 2:**
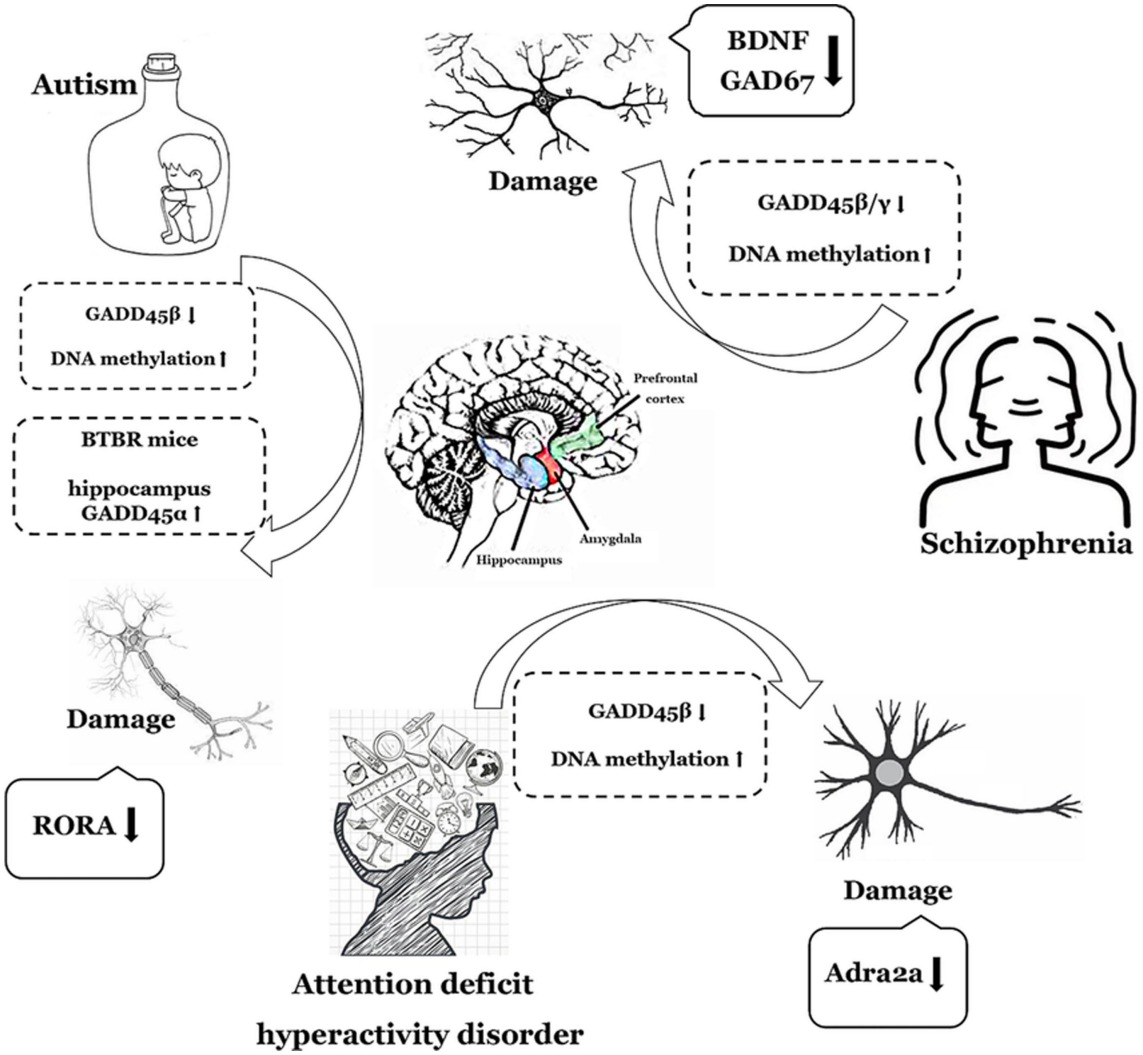
The functions of GADD45 family in neurodevelopmental disorders. The depiction employs a monochromatic line graph to delineate specific neurological disorder types such as autism (ASD), Schizophrenia (SCZ), and attention deficit hyperactivity disorder (ADHD). Adjacent to each neurological disorder, it illustrates the expression levels of GADD45 family members, DNA methylation levels, and the associations with targeted genes related to these disorders, elucidating their respective upregulation (↑) or downregulation (↓) in expression levels. RORA, retinoic acid related-orphan receptor A; BDNF, brain-derived neurotrophic factor.

In summary, members of the GADD45 gene family exhibit abnormally low expression in both ASD and SCZ, with GADD45β also displaying aberrantly low expression in ADHD. While GADD45α may potentially demonstrate anomalous low expression in ASD, further clinical data are imperative for validation. Figure 2 and Table 1 summarize the expression profiles of GADD45 family members in these three disorders.

**Table 1:** Expression of the GADD45 family in ASD, SCZ and ADHD patients and various models.

Diseases	Subject	Model	GADD45 expression	Methylation or demethylation	References
ASD	Patients	N/A	GADD45β↓ cerebral cortex	N/A	Garbett et al. (2008)
	Patients	N/A	GADD45β↓ cerebral cortex	RORA gene methylation↑	Nguyen et al. (2010)
	Patients	N/A	GADD45α↓ peripheral blood T cells	N/A	Tassano et al. (2015)
	Mice	BTBR	GADD45α/γ↑ hippocampus	N/A	Leach et al. (2012)
	Mice	Situational fear conditioning and situational exposure	GADD45β↑ hippocampus	DNA demethylation ↑	Leach et al. (2012)
	Mice	Situational fear conditioning	GADD45β/γ ↑ C1 area	N/A	Sultan et al. (2012)
SCZ	Patients	N/A	GADD45β↓ prefrontal cortex cells	BDNF gene methylation ↑	Gavin et al. (2012)
	Mice	LY379268 i.p.	GADD45β/γ↑ frontal cortex	BDNF and GAD67 gene methylation ↓	Matrisciano et al. (2011)
ADHD	Rat	GADD45β knockdown	GADD45β↓ neonatal rat amygdala	Adra2a promoter methylation↑	Kigar et al. (2015)

Autism spectrum disorders, ASD; Schizophrenia, SCZ; Attention deficit hyperactivity disorder, ADHD; LY379268, mGlu2/3R agonist; BTBR, Black and tan, brachyuric; BDNF, Brain-derived neurotrophic factor; GAD67, Glutamate decarboxylase67; RORA, Retinoic acid related-orphan receptor a.

## 3 GADD45 family in neurodegenerative disorders

Neurodegenerative disorders constitute a category of maladies characterized by the gradual impairment of the nervous system, resulting in apoptosis or functional compromise of neural cells, ultimately impinging upon the normal operation of the nervous system. These conditions typically manifest as chronic, progressively unfolding pathologies that ultimately severely impact an individual’s day-to-day existence and overall quality of life. Neurodegenerative diseases encompass various types, including AD, PD, ALS, among others. Research indicates that GADD45β may be present in neurons, serving as a neuroprotective factor to counteract neuronal damage and aging (Brown et al., 2022). Furthermore, proteins of the GADD45 family may also be associated with the development and plasticity of the nervous system (Brito et al., 2022). Subsequently, we will comprehensively explore the impact of the GADD45 family through DNA methylation on common neurodegenerative diseases such as AD, PD, and ALS, and summarize and analyze their potential signaling pathways and expression patterns of the GADD45 family.

### 3.1 GADD45 family and AD

AD typically presents itself as a progressive manifestation of cognitive impediment and memory loss, standing as the most prevalent geriatric dementia. Age emerges as the foremost pivotal factor in the onset of AD (Zhao et al., 2020). A The pathogenesis of AD exhibits heterogeneity, encompassing amyloid plaques (Depp and Sun, 2023), neurofibrillary tangles (Otero-Garcia et al., 2022), oxidative stress (Ton and Campagnaro, 2020), and aberrant DNA methylation (An et al., 2019). DNA methylation, functioning as a crucial process in gene expression regulation, intricately engages in the modulation of numerous genes associated with the occurrence and progression of AD (Li et al., 2021).

The overall decrease in DNA methylation levels in the cerebral cortex of AD patients correlates inversely with neurofibrillary tangle formation, a hallmark intracellular pathology in AD (Poon et al., 2020). Research investigating alterations in DNA methylation levels throughout the human aging process has unveiled that the entire blood methylome could serve as a potential biomarker for age-related disorders (Hannum et al., 2013; Unnikrishnan et al., 2019). Given the impact of GADD45β on DNA methylation within the epigenetic regulatory framework, the GADD45 family and its associated DNA demethylation modulators may emerge as a second class of targets for pharmacological interventions in neural epigenetic therapeutics (Fuso and Scarpa, 2011; Sultan et al., 2012).

Exposure to amyloid β-peptide (Aβ) in the human neuronal precursor NT2 cell line induces DNA damage, leading to robust expression of GADD45α (Santiard-Baron et al., 2001). This suggests the heightened sensitivity of GADD45α to the genetic toxic stress of AD and its mediation of the DNA damage repair process. Research reveals an elevated expression level of GADD45α in damaged neurons of AD patients’ brains (Torp et al., 1998). These findings suggest that GADD45α is involved in regulating stress responses associated with DNA damage in AD. Furthermore, in the hippocampal tissue of early-stage AD mice models (APP/PS1), there is a downregulation of GADD45α expression, while in the later stages of AD mice, there is an upregulation of GADD45α expression (Liu et al., 2019). This suggests variations in the expression levels of GADD45α during different stages of the AD progression. The rs17070145 variant of the *WWC1* gene, encoding the kidney and brain expressed protein (KIBRA), increases the expression of GADD45β in the human dentate gyrus, thereby promoting the risk of AD occurrence (Piras et al., 2017). In *in vivo* and *in vitro* experiments, glutamate induction in murine hippocampal neuronal cells (HT22) and rat hippocampal tissue elicits an oxidative stress response. The results reveal an elevation in both mRNA and protein levels of GADD45α, while the knockout of the p53 gene inhibits this increase (Choi et al., 2011). In the *Drosophila melanogaster* nervous system, the homologous gene of GADD45α (D-GADD45) exhibits decreased expression with age, and overexpression of this gene extends lifespan (Plyusnina et al., 2011; Moskalev et al., 2012; Plyusnina et al., 2012). This suggests that GADD45α may function through analogous DNA damage repair mechanisms, including MAPK cascades, apoptotic pathways, and oxidative damage signaling. Hence, we have grounds to believe that GADD45 proteins may exert an influence on age-related epigenetic changes, subsequently impacting neurodegenerative diseases associated with aging. However, further research is imperative to delineate the potential contributions of GADD45 genes to age-related epigenetic aberrations.

### 3.2 GADD45 family and PD

PD typically manifests as motor disturbances, predominantly impacting the motor control of the central nervous system. As one of the most prevalent neurological motor disorders globally, the principal pathophysiological mechanisms of PD involve the impairment of DA neurons (Song et al., 2022). These neurons are situated within the substantia nigra and locus coeruleus in the brain, responsible for regulating movement and coordinating muscular activities. The DA neurons in individuals with PD undergo progressive damage or demise, resulting in a reduction in DA levels within the organism (He et al., 2023). Consequently, clinically, PD patients often exhibit manifestations of DA deficiency.

Clinical research reveals that the levels of GADD45β mRNA in the substantia nigra of male PD patients are significantly elevated compared to the control group (Yang et al., 2016). Following 6-OHDA treatment, human male M17 cells exhibit a notable increase in the expression of the Sex-determining region of Y (SRY) gene and GADD45γ on the Y chromosome. The GADD45γ/p38-MAPK/GATA signaling cascade actively activates the SRY promoter (Czech et al., 2014). Moreover, heterochromatin disassembly stands as a key hallmark of aging, and the DNA methylation status currently serves as a primary molecular predictor of chronological age. Treatment of human DA neuroblastoma M17 cells with MPP+ induces a time- and dose-dependent upregulation of GADD45α expression. Subsequently, GADD45α binds to the c-Jun promoter, orchestrating the regulation of cellular proliferation and apoptosis processes. These findings substantiate the protective role of GADD45α against MPP+ toxicity in M17 DA neuroblastoma cells (Wang et al., 2014).

In PC12 cells treated with zinc (Zn^2+^) and DA, there is an elevation in both the mRNA and protein levels of GADD45β. Furthermore, the MAPK p38 and JNK signaling pathways exhibit cross-talk with GADD45β. In *in vivo* experiments, intracerebral infusion of Zn^2+^ and DA in male C57BL/6 mice results in increased levels of GADD45β protein and phosphorylation of p38 in the substantia nigra. Hence, the augmentation of GADD45β may confer benefits to individuals with AD (Park et al., 2016). Overexpression of GADD45β in wild-type Swiss mice leads to dysregulation of DNA methylation, wherein DNA methylation-induced chromatin structural changes and DNA damage emerge as significant contributors to the pathogenic mechanisms underlying DA neuron degeneration, potentially culminating in PD pathology (Ravel-Godreuil et al., 2021). Drosophila exposed to the herbicide paraquat exhibit downregulation of GADD45 expression in DA neurons, accompanied by a decrease in fly survival rates implying a neuroprotective role of GADD45 against paraquat- induced neurotoxicity (Maitra et al., 2019).

### 3.3 GADD45 family and ALS

ALS, also known as Lou Gehrig’s disease, is a debilitating motor neuron disorder characterized by the gradual degeneration and impairment of both upper and lower motor neurons (Feldman et al., 2022). This condition leads to progressive muscle atrophy and the inability to perform normal movements, ultimately affecting muscular control and coordination. The average life expectancy post-diagnosis for individuals with ALS is approximately 2–5 years (Hardiman et al., 2017). In confirmed ALS cases, around 90–95% are classified as sporadic ALS (sALS), while approximately 5–10% are familial ALS (fALS), indicating a complex interplay of genetic and environmental factors in the etiology of the disease. It is crucial to emphasize that there is currently no definitive method for preventing or curing ALS. Consequently, a comprehensive understanding of the pathogenesis and progression of ALS and the quest for more efficacious therapeutic strategies hold paramount clinical significance.

One of the most prevalent genetic factors associated with ALS is the hexanucleotide repeat expansion GGGGCC (G4C2) in the 9th chromosome open reading frame 72 gene (C9ORF72) (Rohrer et al., 2015). Clinical observations indicate that in induced pluripotent stem cell (iPSC) lines differentiated into motor neurons from C9ORF72 ALS patients, there is an elevation in the levels of DNA damage markers γH2AX, ATR, GADD45α, and p53 in response to oxidative stress (Lopez-Gonzalez et al., 2016). GADD45α emerges as the most significantly differentially expressed transcript in the denervated gastrocnemius muscle of acutely denervated Swiss mice and maintains induced expression for up to 3 months post-denervation (Ehmsen et al., 2021). Comparative analysis of denervated skeletal muscle samples from patients with traumatic nerve injury or acute flaccid myelitis reveals a significant increase in GADD45α expression in denervated muscles compared to intact control muscles (Ehmsen et al., 2021). In the SOD1G86R mouse model, one of the existing ALS animal models, high-density oligonucleotide microarray analysis of denervated muscles from G86R mice indicates an upregulation of GADD45α (Gonzalez de Aguilar et al., 2008).

**Figure 3:**
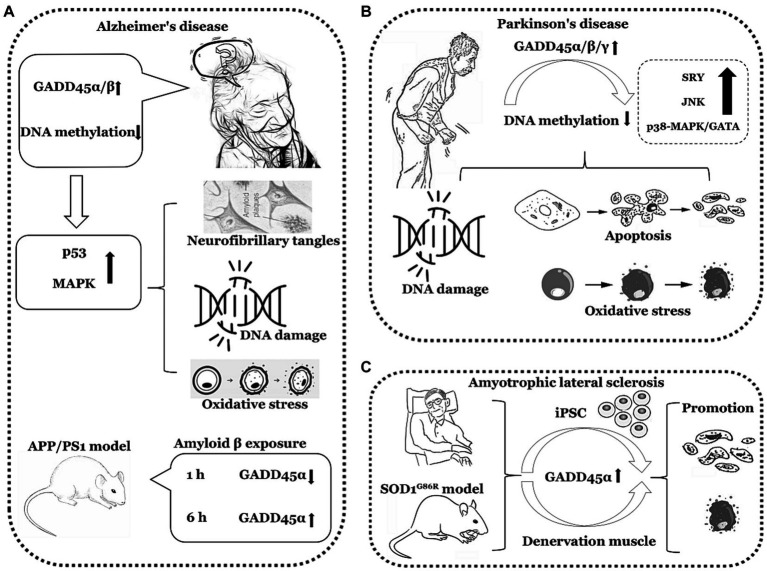
The functions of GADD45 family in neurodegenerative disorders. **(A)** Expression of GADD45α/β increased in Alzheimer’s disease (AD). **(B)** Expression of GADD45α/β/γ increased in Parkinson’s disease (PD). **(C)** Expression of GADD45α increased in amyotrophic lateral sclerosis (ALS). SRY, sex-determining region Y. The image herein employs a monochromatic linear representation to delineate specific neurodegenerative disorders, such as AD, PD, and ALS. Adjacent to each neurological disorder, it illustrates explorations in clinical or foundational research regarding the expression levels of GADD45 family members, DNA methylation levels, and the relationships with targeted genes associated with these diseases, elucidating their respective upregulation (↑) or downregulation (↓) in expression levels.

In summary, the expression of GADD45α and GADD45β is elevated in AD and PD, the expression of GADD45α is also increased in ALS. However, clinical data directly assessing the GADD45 family in AD, PD, and ALS remain limited, necessitating further validation through additional clinical data. The comprehensive summary of GADD45 family members in these three diseases is depicted in Figure 3 and Table 2.

**Table 2:** Expression of the GADD45 family in AD, PD and ALS patients and various models.

Diseases	Subject	Model	GADD45 expression	Methylation or demethylation	References
AD	NT2 cells	Amyloid β exposure	GADD45α↑	N/A	Santiard-Baron et al. (2001)
	Mice	APP/PS1 model	1 h GADD45α↓ hippocampus 6 h GADD45α↑ hippocampus	N/A	Liu et al. (2019)
	Patients	rs17070145-T mutant	GADD45β↑ dentate gyrus	N/A	Piras et al. (2017)
	HT22 cells	Glutamate induced	GADD45α↑	N/A	Choi et al. (2011)
	Rat	Glutamate induced	GADD45α↑	N/A	Choi et al. (2011)
	Fruit fly	N/A	D-GADD45↓	N/A	Plyusnina et al. (2011)
PD	Patients	N/A	GADD45β↑ substantia nigra	N/A	Yang et al. (2016)
	M17 cells	6-OHDA induced	GADD45γ↑	N/A	Czech et al. (2014)
	M17 cells	MPP+ induced	GADD45α↑	N/A	Wang et al. (2014)
	PC12 cells	Zn^2+^ and DA induced	GADD45β↑	N/A	Park et al. (2016)
	Mice	Zn^2+^ and DA induced	GADD45β↑ substantia nigra	N/A	Park et al. (2016)
	Mice	GADD45β overexpression	GADD45β↑ brain	DNA methylation disorder	Ravel-Godreuil et al. (2021)
ALS	Patients	C9ORF72	GADD45α↑ iPSC	N/A	Lopez-Gonzalez et al. (2016)
	Patients	Nerve injury or acute flaccid myelitis	GADD45α↑denervation muscle	N/A	Ehmsen et al. (2021)
	Mice	SOD1^G86R^	GADD45α↑denervation muscle	N/A	Gonzalez de Aguilar et al. (2008)

AD, Alzheimer’s disease; PD, Parkinson’s disease; ALS, Amyotrophic lateral sclerosis; 6-OHDA, 6-hydroxydopamine; DA, Dopamine; iPSC, Induced pluripotent stem cells; MPP+, 1-Methyl-4-phenyl-1,2,3,6-tetrahydropyridine; SOD1, Superoxide dismutase.

## 4 GADD45 family in neuropsychiatric disorders

Neuropsychiatric disorders constitute a spectrum of illnesses involving multifaceted issues related to cognition, emotion, behavior, and social functioning, encompassing abnormalities in both the neurological and psychiatric domains. Different types of disorders exhibit distinct pathological features; for instance, MDD patients may manifest an imbalance in neurotransmitters such as serotonin and DA (Bekhbat et al., 2022). PTSD patients may exhibit elevated levels of corticotropin-releasing factor (CRF), indicating dysregulation in the hypothalamic- pituitary–adrenal axis neuroendocrine regulatory function (Bremner et al., 1997). Research indicates that sleep disturbances associated with traumatic brain injury and increased gene methylation levels linked to depression are correlated, alongside a reduction in gene expression (Haghighi et al., 2015). The GADD45 family, serving as facilitators of DNA demethylation, may potentially influence the development of neuropsychiatric disorders by modulating the DNA methylation levels of specific genes. In the subsequent discussion, we will comprehensively explore the impact of the GADD45 family on MDD, PTSD, BD, and ADD in neuropsychiatric disorders, summarizing their expression patterns and potential signaling pathways.

### 4.1 GADD45 family and MDD

The primary clinical features of MDD encompass persistent, despondent mood, a loss of interest or pleasure in daily activities, and may be accompanied by other symptoms such as fatigue, sleep disturbances, difficulty concentrating, and negative self-thinking. The pathological characteristics of MDD include abnormalities in neural network function, particularly within regions involved in emotion regulation and cognitive control (Chen et al., 2021). Furthermore, there is an imbalance in neurotransmitters, such as aberrant expression of serotonin and norepinephrine (Naoi et al., 2018). Excessive activation of the inflammatory response in the immune system is also a notable pathological feature (Dantzer et al., 2008). Additionally, there is evidence of familial clustering and a genetic predisposition phenomenon associated with MDD (Shadrina et al., 2018).

In the Flinder Sensitive Line (FSL) genetic rat model of depression, untreated rats exhibited a decrease in GADD45β mRNA levels in the prefrontal cortex (Melas et al., 2012). Yin et al. (2021) found a significant correlation between the downregulation of the GADD45β gene and depressive-like behavior after ischemic stroke. GADD45β may play a positive role in the remodeling activity of hippocampal neurons in adult rats through the BDNF signaling pathway, thereby alleviating post-stroke depression. This suggests the involvement of GADD45β in the induction of antidepressant-like activities. In a study using the Unpredictable Chronic Mild Stress (UCMS) model to induce depressive-like behavior in mice, researchers observed a significant decrease in the expression of GADD45α, GADD45β, and GADD45γ in the hippocampus and prefrontal cortex of UCMS mice. Additionally, there was a reduction in the transcription of the mood disorder susceptibility gene Arc. In the hypothalamus, the expression of GADD45α and γ remained unchanged, while the expression of GADD45β increased (Grassi et al., 2017). Following depolarization of mature hippocampal neurons induced by KCl *in vitro* in C57BL/6J mice, there is an increase in the transcription of DNA demethylases, as well as elevated release of BDNF and transforming growth factor beta (TGFB). Concurrently, the expression levels of GADD45β and GADD45γ mRNA significantly increase. Inhibitor blockade experiments further confirm that TGFB and BDNF negatively regulate the expression of GADD45 family members providing additional evidence that the GADD45 family modulates the expression of downstream target genes BDNF and TGFB through the facilitation of the DNA demethylation process (Grassi et al., 2017). The expression levels of GADD45 genes are lower under conditions of depression, and analyzing DNA methylation patterns and the transcription of specific genes in related cell subpopulations holds crucial clinical significance. Electroconvulsive therapy (ECT) is an effective method for treating MDD (Azis et al., 2019). In depressive patients undergoing a single session of ECT, there is a rapid upregulation of Gadd45β and Gadd45γ mRNA in the dentate gyrus tissue, promoting the DNA demethylation of the BDNF gene (Ma et al., 2009). Following ECT treatment, gene expression profiling of granule cells in the rat hippocampal dentate gyrus reveals upregulation of BDNF, neuropeptide Y, and Gadd45β (Ploski et al., 2006). Metformin (Met), a compound known as dimethylbiguanide, represents an agent that fosters hippocampal neurogenesis for the prevention and treatment of MDD. *In vitro* cellular experiments have elucidated that Met facilitates the expression levels of GADD45γ mRNA in hippocampal neurons of C57BL/6 mice. It appears to exert no significant impact on the expression levels of GADD45α and GADD45β. Concurrently, during the process of neuronal differentiation induced by Met, GADD45γ plays a role in promoting DNA demethylation. The downregulation of GADD45γ in the hippocampus of mice results in cognitive impairments and depressive-like behaviors (Zhang et al., 2022). Consequently, it is hypothesized that the GADD45 family exhibits abnormally low expression in MDD, leading to the manifestation of depressive behaviors in patients through the facilitation of DNA demethylation of downstream specific target genes such as BDNF, TGFB, or neuropeptide Y.

### 4.2 GADD45 family and PTSD

PTSD is a psychopathological condition intricately linked to severe traumatic incidents in one’s past. Its primary features manifest as a continuum of stress-induced symptoms, notably characterized by recurrent re-experiencing, avoidance behaviors, emotional numbness, heightened vigilance, and intense emotional oscillations following exposure to the traumatic event. Clinical investigations underscore a noteworthy diminution, in the DNA methylation levels of specific genes associated with the immune system within the peripheral blood of individuals afflicted with PTSD (Uddin et al., 2010). Furthermore, there is an enrichment of oxidative stress and inflammation-associated signaling pathways. Additionally, a discernible correlation exists between the activity of the GADD45 signaling pathway and the secondary progression of affliction in PTSD patients (Kang and Lee, 2021). Research posits an augmented susceptibility to immune-related disorders in the wake of PTSD, encompassing cardiovascular ailments, diabetes, atherosclerosis, and autoimmune conditions (Levine et al., 2014). Females afflicted with chronic PTSD exhibit elevated levels of pro-inflammatory cytokines, thereby precipitating endothelial dysfunction (Sumner et al., 2017). Both animal and human studies converge in indicating a consequential association between robust inflammatory activity and the progression of PTSD (Dantzer et al., 2008; Raison et al., 2013). Pro-inflammatory cytokines circulating in the periphery have the capacity to traverse the blood–brain barrier, inciting neuroinflammation (Banks et al., 2002). This elucidates the profound correlation between the GADD45 family, oxidative stress, and the RORA gene rs8042149 variant in PTSD. Individuals harboring the RORA gene rs8042149 mutation are predisposed to a heightened susceptibility to PTSD (Logue et al., 2013). Liebermann et al. discerned that GADD45α and GADD45β within murine peritoneal granulocytes, by modulating the expression levels of the p38 and JNK signals, play a facilitating role in innate myeloid immune functions such as reactive oxygen species generation, phagocytosis, and adhesion (Salerno et al., 2012). The aforementioned biological processes involve immune function, inflammatory activity, and the expression levels of specific genes, all of which are associated with the expression levels of GADD45 family members. Hence, it is plausible to infer that GADD45 exhibits aberrant expression in PTSD, with a higher occurrence of elevated GADD45 expression.

### 4.3 GADD45 family and BD

BD, also known as manic-depressive illness, is characterized by cyclic emotional fluctuations, encompassing periods of elevated mood (manic episodes) and periods of diminished mood (depressive episodes). The pathological underpinnings of this disorder primarily implicate imbalances in neurotransmitters. During manic episodes, DA levels may ascend, accompanied by heightened neural activity in the amygdala and prefrontal cortex, instigating elevated mood and impulsive behaviors. Conversely, in depressive episodes, DA levels may decline, concomitant with diminished neural activity in the prefrontal cortex and amygdala, fostering lowered mood and self-deprecating thought patterns.

Through exon sequencing of lymphoblastoid cells from individuals with BD, exceedingly rare and heterozygous protein-damaging variants were identified in eight brain-related genes (IQUB, JMJD1C, GADD45α, GOLGB1, PLSCR5, VRK2, MESDC2, and FGY). Proteins encoded by these genes play pivotal regulatory roles in crucial pathways such as ERK/MAPK and CREB, governing cell proliferation, differentiation, and apoptosis, which are essential for neuronal and synaptic plasticity, cognition, emotional regulation, and chronic stress responses (Kerner et al., 2013). Within the Inferior Parietal Lobule (IPL) of BD patients, a notably elevated methylation level was detected in the GAD67 promoter region, concomitant with a decrease in GAD67 mRNA expression (Dong et al., 2012). This discovery underscores the potential role of DNA demethylation mechanisms in the pathophysiology of BD. Considering the previous research, the upregulation of GADD45β and GADD45γ can enhance the expression levels of GAD67 (Matrisciano et al., 2011). Consequently, it can be inferred that the expression levels of GADD45 in the IPL of BD patients are diminished. Valproic acid (VPA), a short-chain fatty acid derivative, is employed as a mood stabilizer in the treatment of BD (Cipriani et al., 2013). Following VPA treatment, elevated expression levels of GADD45α were observed in neuroblastoma cells (N1E-115), activating downstream JNK cascades, thereby influencing neuronal synaptic growth (Yamauchi et al., 2007). Studies have indicated that both mRNA and protein expression levels of GADD45β increased in the nucleus of prefrontal cortex and cortical pyramidal neurons in mice treated with VPA (Guidotti et al., 2011). This suggests that VPA, along with antipsychotic medications, may exert therapeutic effects by modulating the DNA demethylation levels of relevant genes, altering the expression levels or activity of GADD45.

### 4.4 GADD45 family and ADD

The primary hallmark of ADD is an excessive dependence on and an uncontrollable craving for a particular substance or activity, thereby adversely affecting individual well-being and health. ADD encompasses substance use disorders (such as alcohol, drugs, nicotine, etc.) and behavioral addictions (such as gambling, food, gaming, etc.), with the pathological features varying based on the type of addiction (e.g., substances, alcohol, gambling). Alterations in neurotransmitter levels, particularly DA, constitute a pivotal pathological feature of ADD, given that this neurotransmitter enhances the activity of the reward system (Leyton and Vezina, 2014). Addictive substances or behaviors can activate the brain’s reward pathways, inducing feelings of pleasure and reward, thereby fostering the misuse of addictive substances or behaviors. Furthermore, prolonged addiction may lead to alterations in the tolerance and adaptability of the reward pathways, necessitating increased or more potent stimuli to elicit the same sense of pleasure.

Clinical research indicates an increased susceptibility to alcohol dependence in individuals who have experienced adverse childhood events (CA), possibly attributed to CA-induced epigenetic alterations. CA can induce specific methylation changes in the promoter regions of certain genes (ALDH1A1, OPRL1, and RGS19), thereby influencing gene transcription levels (Zhang et al., 2013). Following stimulant exposure, the methylation changes in specific gene promoters exhibit a significant correlation with drug abuse behavior and the likelihood of physiological adaptation (Wang et al., 2012). The acute administration of cocaine can induce a rapid upregulation of GADD45β mRNA in the ventral tegmental area of SD rats. Conversely, knocking down the GADD45β gene can impede cocaine-induced behavioral preferences, reduce gene expression levels associated with stimulant addiction, and halt the induction of DA receptor D1 (DRD1) in relation to these genes (Zipperly et al., 2021). From a genetic perspective, this insight is invaluable for understanding gambling addiction and other behavioral addictions. It aids in distinguishing specific psychiatric disease phenotypes, thereby holding significant implications for the clinical diagnosis and treatment of mental disorders.

**Figure 4:**
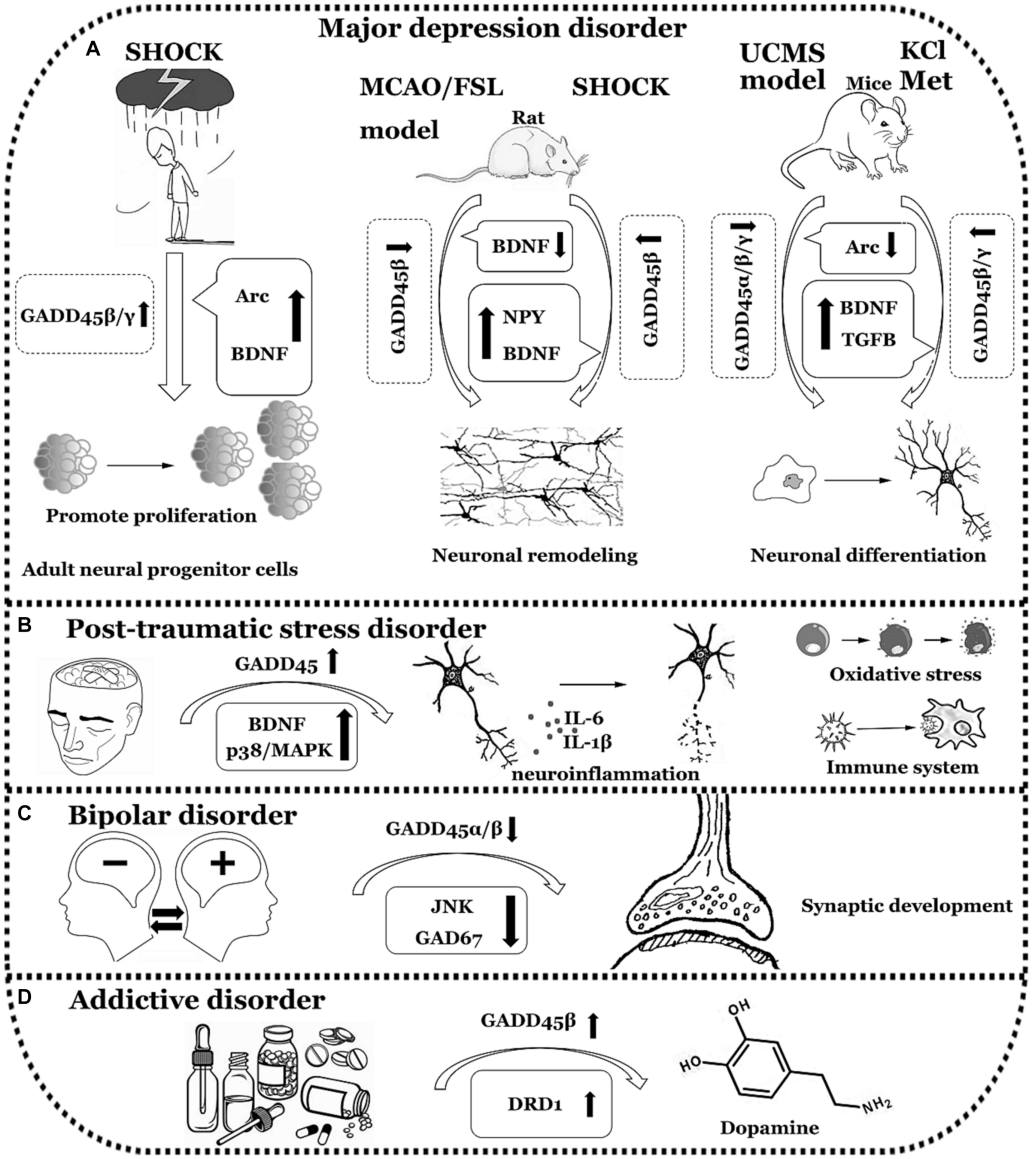
The functions of GADD45 family in neuropsychiatric disorders. **(A)** Expression of GADD45α/β/γ decreased in Major depression disorder (MDD). **(B)** Expression of GADD45 increased in Post-traumatic stress disorder (PTSD). **(C)** Expression of GADD45β decreased in Bipolar disorder (BD). **(D)** Expression of GADD45β increased in Addictive disorder (ADD). UCMS, unpredictable chronic mild stress; Arc, activity regulated cytoskeletal-associated protein; Met, metformin; FSL, flinders sensitive line; MCAO, Middle cerebral artery occlusion; DRD1, dopamine receptor 1. The diagram illustrates specific neurological disorders such as MDD, PTSD, BD, and ADD using a simple black-and-white line graph. Adjacent to each neurological disorder, it depicts explorations in clinical or foundational research concerning the expression levels of GADD45 family members, DNA methylation levels, and the relationships with targeted genes associated with these diseases, indicating their respective upregulation (↑) or downregulation (↓) in expression levels.

In summary, individuals with MDD exhibit a decrease in the expression levels of GADD45β and GADD45γ. Similarly, both PTSD and BD patients display reduced expression of GADD45β. In contrast, individuals with ADD show an elevation in GADD45β expression. Nevertheless, direct data confirming the relationship between the GADD45 family and ADD is lacking, necessitating further validation through clinical data. The comprehensive summary of GADD45 family members in these four disorders can be observed in Figure 4 and Table 3.

**Table 3:** Expression of the GADD45 family in MDD, PTSD, BD and AD patients and various models.

Diseases	Subject	Model	GADD45 expression	Methylation or demethylation	References
MDD	Rat	FSL model	GADD45β↓ prefrontal cortex	DNA hypermethylation	Melas et al. (2012)
	Rat	MCAO model+GADD45β knockdown	GADD45β↓	N/A	Yin et al. (2021)
	Mice	UCMS exposure	GADD45α/β/γ↓ hippocampus and prefrontal cortex	N/A	Grassi et al. (2017)
	Mice	KCl induced	GADD45β/γ↑ mature hippocampal neurons	BDNF and TGFB DNA demethylation↑	Grassi et al. (2017)
	Patients	ECT treatment	GADD45β/γ↑dentate gyrus	BDNF DNA demethylation↑	Ma et al. (2009)
	Rat	ECT treatment	GADD45β↑dentate gyrus	N/A	Ploski et al. (2006)
	Mice	Metformin treatment	GADD45γ↑hippocampal neuron	DNA demethylation↑	Zhang et al. (2022)
PTSD	Patients	N/A	GADD45↑	N/A	Kang and Lee (2021)
	Patients	N/A	N/A	DNA methylation↓	Uddin et al. (2010)
BD	Patients	N/A	GADD45β↓ IPL	GAD67 DNA methylation ↑	Dong et al. (2012)
	N1E-115 cells	VPA	GADD45α↑	N/A	Yamauchi et al. (2007)
	Mice	VPA	GADD45 β↑ frontal cortex	N/A	Guidotti et al. (2011)
ADD	Rat	Cocaine	GADD45β↑ nucleus accumbens	DRD1 DNA demethylation ↑	Zipperly et al. (2021)

MDD, Major depression disorder; PTSD, Post-traumatic stress disorder; BD, Bipolar disorder; ADD, Addictive disorder; FSL, Flinders sensitive line; MCAO, Middle cerebral artery occlusion; UCMS, Unpredictable chronic mild stress; ECT, Electroconvulsive therapy; IPL, Inferior parietal lobule; VPA, Valproic acid.

## 5 Conclusion and prospects

The GADD45 family exhibits widespread expression throughout the central nervous system, playing a pivotal and indispensable role in various neurological disorders. Among these, GADD45α shows increased expression in ASD, AD, PD, and ALS, while decreased expression in BD. GADD45α significantly impairs neuronal morphology, reduces the complexity of neuronal synapses, and disrupts neuronal differentiation, suggesting a crucial role for GADD45α in the development of neural circuits and making it a potential target for ASD. GADD45α is involved in regulating stress responses associated with DNA damage and exhibits spatiotemporal specific expression during the progression of AD, we suggest that GADD45α may participate in the regulation of AD pathology through analogous DNA damage repair mechanisms, including MAPK cascades, apoptotic pathways, and oxidative damage signaling. Moreover, GADD45α may exert neuroprotective effects through the regulation of p38-MAPK/GATA, SRY, and JNK pathway expression, thereby mitigating MPP + -induced DNA damage and oxidative stress toxicity, indicating a potential role in PD. GADD45β displays differential expression in the majority of central nervous system disorders. Specifically, GADD45β demonstrates upregulation in PD, AD, and ADD, while displaying downregulation in ASD, SCZ, ADHD, MDD, PTSD, and BD. Furthermore, the downstream pathological pathways associated with GADD45β in these studies exhibit some inconsistency, encompassing oxidative stress, DNA damage, and inflammatory responses. This variability underscores the multifaceted biological functionality of GADD45β in the development of central nervous system disorders, with this diversity closely linked to its role in DNA methylation. Notably, GADD45γ demonstrates decreased expression in SCZ, while exhibiting increased expression in PD and MDD. The upregulation of GADD45γ within the hippocampal dentate gyrus contributes to adaptive responses by facilitating BDNF demethylation; conversely, downregulation of GADD45γ within the hippocampus leads to cognitive impairments and depressive-like behaviors. Currently, there is a paucity of research investigating the underlying mechanisms of GADD45 family, particularly focusing on GADD45α and GADD45γ, in relation to neurological disorders. Therefore, the exploration of GADD45 family in this field holds great promise.

This review provided additional insights into the limited research findings on GADD45 family, particularly focusing on the expression and mechanism of action of GADD45α and GADD45γ in neurological disorders, and further explored the correlation between the GADD45 family and less-studied or even unstudied conditions such as PTSD and ADD, thereby addressing knowledge gaps concerning the involvement of the GADD45 family in central nervous system disorders. In light of this, future research endeavors should focus on analyzing the expression patterns of the GADD45 family genes, DNA methylation levels, and potential downstream pathway alterations. Establishing connections between genes and phenotypes will unveil novel pathogenic genes and pathways associated with central nervous system disorders. This will, in turn, furnish reliable genetic-level clinical adjunct evidence, facilitating precise prevention and treatment for central nervous system disorders. Additionally, it will aid researchers in identifying new therapeutic targets and disease biomarkers.

## Author contributions

MH: Formal analysis, Funding acquisition, Writing – original draft. JW: Formal analysis, Funding acquisition, Writing – original draft. WL: Formal analysis, Writing – review & editing. HZ: Writing – review & editing.

## Footnotes

^1^https://www.uniprot.org/
